# COVID-19’s impact on neglected pharmaceutical staff: wake-up call for needed research

**DOI:** 10.1186/s40545-021-00376-x

**Published:** 2021-11-04

**Authors:** Richard Antony Powell, Shivali Lakhani, Marsha Alter, Steven Guan, Jehanita Jesuthasan, Dasha Nicholls

**Affiliations:** 1 NIHR ARC Northwest London, London, England; 2 Department of Primary Care and Public Health, Faculty of Medicine, School of Public Health, Imperial College London, Sir Alexander Fleming Building, South Kensington Campus, London, SW7 2AZ England; 3 Ethnicity and Health Unit, School of Public Health, Imperial College London, London, England; 4 Middlesex Pharmaceutical Group, London, England; 5 Department of Brain Sciences, Imperial College London, London, England

**Keywords:** COVID-19, Community pharmacy, Mental health, Pandemic, Pharmacists, Professional roles

## Abstract

Discussion of the necessity of the compulsory vaccination of UK patient-facing care workers as an employment conditionality has deflected from the initial and ongoing impact of Coronavirus disease on relatively neglected occupational groups themselves, including community pharmacists. This commentary highlights the relative lack of research investigating the mental health and wellbeing impact of the pandemic on this occupational group in England and urges further study of their needs and experiences to inform evidence-based supportive psychological interventions.

## Main text

Valid discussion of the necessity and aptness of the compulsory vaccination of UK patient-facing care workers as an employment conditionality [1–3] has deflected from the initial and ongoing impact of Coronavirus disease (COVID-19) on relatively neglected occupational groups themselves.

Evidence of the severity of detrimental effect of the virus, including morbidity and mortality, on secondary care service personnel is increasing [4, 5]. Growing data exist on the short-term psychological sequelae of the pandemic in its first and second waves among acute, hospital-based doctors and nursing staff [6–9]. However, the impact of the pandemic on the mental health and wellbeing of secondary care support staff (e.g., administrators, cleaners) and community-based staff (e.g., primary care physicians, social care staff), on which services rely, have been comparatively overlooked [10].

Community pharmacists comprise one such occupational group. Working as frontline healthcare workers, pharmacists have acted as the first point of patient contact, triaging patients, disseminating information, managing medication shortages, and experiencing patient harassment, with resultant increased workload and potential mental health consequences [11].

International research has been conducted exploring the impact of the pandemic on the pharmacy profession, as part of wider healthcare worker studies [12–14] and as a dedicated professional group [11, 15, 16]. One of the most ambitious studies covered over 500 pharmacists in 31 Commonwealth countries [17]. It found the majority reported being at least somewhat worried (90%), and over 65% very or extremely worried, about COVID-19’s impact personally and professionally, and nearly two-thirds found it somewhat or very difficult to work effectively during the pandemic [17].

Despite being one of the four pillars of the country’s primary care system—numbering over 11,500 pharmacies across England [18], and nearly 56,000 pharmacists and over 24,000 pharmacy technicians across the UK [19]—incomparable research has been conducted among English pharmacists. One of the few such studies was the nationwide “Workforce Mental Health and Wellbeing Survey” conducted by the Royal Pharmaceutical Society (RPS) with the Pharmacist Support charity during the pandemic’s first wave [20]. The study results were startling: nearly a third (31%) of pharmacists reported that COVID-19 had impacted their mental health significantly but that 44% felt uncomfortable accessing employer- and NHS-funded occupational health services, citing confidentiality and trust, stigma and judgement, and their potential to harm their careers. Among this group not seeking support, those working in the community were most reluctant (51%), compared to those in general practice (46%) and hospital (31%).

In relation to their work, 72% reported the pandemic had negatively affected their mental health and wellbeing, manifesting as increased service demand, inadequate staffing, long hours and a lack of breaks and time off. Unsurprisingly, the overwhelming majority (89%) scored as being at high risk of burnout, one third (33%) had considered leaving their job, and 34% had considered leaving the profession. However, the most disarming finding was comparing the study results with pre-pandemic 2019 data, when 74% reported negative mental health and wellbeing from their work environment, and 80% scored being at high risk of burnout [21].

The broadly consistent personal and occupational stress and burnout found among English pharmacists appears embedded in the profession’s self-perception of being “little more than glorified retailers,” their role within the healthcare sector often “downplayed and even underplayed” [22]. This stands in contrast to the general public’s positive view of the profession (89% in one study) as “essential” during the pandemic, adapting well to the virus, offering a “good patient service” and going “above and beyond” [23]. It is also linked to the precarious financial health of many pharmacies—with three-quarters anticipated to be loss-making within four years—arising from the 2016 funding cuts they received and the current settlement of 5 years’ flat funding [24].

Lastly, in the post-pandemic landscape facing the NHS, further demands are being placed on community pharmacists to assist with addressing the accumulated backlog of patient appointments and cases. Given their often-easy high street accessibility, pharmacies are well positioned to act as an early diagnostic and advisory entry point to the healthcare system, thereby relieving pressure on general practitioners (GPs). However, an audit recently revealed English pharmacies are already providing 1.1 million unremunerated consultations per week [25], while rising patient numbers and a shortage of GPs threaten to overwhelm the system [26].

## Conclusions

The net result of these multi-factorial pressures, compounded by COVID-19, is a real need for further study of this under-researched group. There is a pressing need to look at the longer-term mental health and wellbeing needs of pharmacists in the post-pandemic environment and its new realities, ideally longitudinally. But it is also imperative to understand how these are inter-related with, and potentially exacerbated by, underlying historical and contemporary personal and professional challenges, be they contractual, financial, and/or occupational burden. It is only by appreciating these needs and experiences that supportive, evidence-based psychological interventions to meet them can be developed and recommended.

## Data Availability

Not applicable.

## Abbreviations

COVID-19: Coronavirus disease
GP: General practitioner
NHS: National Health Service
RPS: Royal Pharmaceutical Society
